# Persistent bone impairment despite long-term control of hyperprolactinemia and hypogonadism in men and women with prolactinomas

**DOI:** 10.1038/s41598-021-84606-x

**Published:** 2021-03-04

**Authors:** Lukas Andereggen, Janine Frey, Robert H. Andres, Markus M. Luedi, Hans Rudolf Widmer, Jürgen Beck, Luigi Mariani, Emanuel Christ

**Affiliations:** 1 Department of Neurosurgery, Neurocenter and Regenerative Neuroscience Cluster, Inselspital, Bern University Hospital, University of Bern, Bern, Switzerland; 2 Department of Endocrinology, Diabetes, and Metabolism, Inselspital, Bern University Hospital, University of Bern, Bern, Switzerland; 3 Department of Anaesthesiology and Pain Medicine, Inselspital, Bern University Hospital, University of Bern, Bern, Switzerland; 4 Department of Neurosurgery, Medical Center, University of Freiburg, Freiburg im Breisgau, Germany; 5 Department of Neurosurgery, University Hospital of Basel, Basel, Switzerland; 6 Division of Endocrinology, Diabetes and Metabolism, Department of Endocrinology, University Hospital of Basel, Petersgraben 4, 4031 Basel, Switzerland; 7 Department of Neurosurgery, Kantonsspital Aarau, Aarau, Switzerland

**Keywords:** Pituitary diseases, CNS cancer

## Abstract

While prolactinoma patients have high bone turnover, current data are inconclusive when it comes to determining whether correction of hyperprolactinemia and associated hypogandism improves osteodensitometric data in men and women over the long term. In a large cohort of including 40 men and 60 women, we studied the long-term impact of prolactinoma treatment on bone mineral density (BMD) in men versus women, assessed adverse effects of a primary surgical or medical approach, and evaluated data for risk factors for impaired BMD at last follow-up using multivariate regression analyses. Median duration of follow-up was 79 months (range 13–408 months). Our data indicate that the prevalence of impaired BMD remained significantly higher in men (37%) than in women (7%, *p* < 0.001), despite the fact that hyperprolactinemia and hypogonadism are under control in the majority of men. We found that persistent hyperprolactinemia and male sex were independent risk factors for long-term bone impairment. Currently, osteoporosis prevention and treatment focus primarily on women, yet special attention to bone loss in men with prolactinomas is advised. Bone impairment as “end organ” reflects the full range of the disease and could become a surrogate marker for the severity of long-lasting hyperprolactinemia and associated hypogonadism.

## Introduction

Impaired bone mineral density (BMD) is associated with post-menopausal women^1–3^, but is often underdiagnosed in men^4–6^. Prolactinoma patients have high bone turnover, impairing BMD^7–9^. Hyperprolactinemia and the associated hypogonadism may cause secondary osteoporosis^10–13^, which has been related to skeletal fragility in both men and women^14, 15^. While some data indicate that hyperprolactinemic subjects do not demonstrate increased fractures despite their low bone density^16^, other studies have reported a higher prevalence of vertebral fractures in particular in postmenopausal women with untreated prolactinomas, compared to patients treated with dopamine agonists (DAs)^15^. However, there is a lack of evidence that normalization of prolactin levels improves BMD or reduces the fracture risk^17^.

Likewise, it remains unclear whether prolactin (PRL) plays an independent role, separate from gonadal status, in the impairment of BMD, and whether controlling both improves bone health^9,14, 18, 19^. While prolactin excess per se may contribute to skeletal fragility^9^, the effects of hyperprolactinemia on gonadal function or on bone might be independent of gonadal function^20^. Namely, normalization of prolactin and restoration of gonadal function might increase bone density, but this has not been associated with normalization of bone mass^21^, or reduction of fracture risk^22^.

We hypothesized that correction of hyperprolactinemia and associated hypogonadism improves osteodensitometric data in both men and women over the long-term. In a large cohort study in a dedicated tertiary referral center, we thus investigated whether prolactinoma treatment has an impact on the prevalence of bone impairment in both sexes over the long-term, and we assessed risk factors for impaired BMD that might guide better-targeted therapies.

## Results

### Patient characteristics at baseline

Between 1997 and 2015, osteodensitometric data were assessed in one hundred prolactinoma patients (40 men, 60 women) at Bern University Hospital at study entry and at long-term follow-up (> 12 months). Patient characteristics are summarized in Table 1.

**Table 1:** Patient characteristics at baseline.

	Men	Women	Total	*p* value
Number of patients, n (%)	40 (40)	60 (60)	100 (100)	
Age at diagnosis in years (mean ± SD)	46.6 ± 14.8	34.3 ± 11.5	39.2 ± 14.2	**< 0.001**
BMI (kg/m^2^ ± SD)	28.7 ± 4.6	25.5 ± 5.6	26.9 ± 5.4	**0.008**
**Treatment, n (ratio)**				
Medical: surgical	28/12 (2:1)	19/41 (1:2)	47/53	**n/a**
Headache, n (%)	17 (43)	12 (20)	29 (29)	**0.02**
**Affected pituitary axes, n (%)**				
Gonadotropin deficiency	16 (84)	44 (92)	60 (90)	**0.39**
Secondary hypothyroidism	4 (13)	4 (7)	8 (9)	**0.45**
Secondary adrenal insufficiency	4 (12)	1 (2)	5 (5)	**0.05**
**Tumor size, n (%)**				
Macroadenoma	32 (80)	21 (35)	53 (53)	**< 0.001**
Microadenoma	8 (20)	39 (65)	47 (47)	**< 0.001**
Tumor invasiveness	32 (80)	5 (9)	37 (39)	**< 0.001**
Prolactin levels in μg/L (median; IQR)	1978.5 (768–6875)	152 (88–268)	252.6 (110–1704)	**< 0.001**
Impaired bone mineral density	11 (28)	1 (2)	12 (12)	**< 0.001**
Follow-up time in months (mean ± SEM)	81.6 ± 9.3	114.2 ± 12.5	101 ± 8.5	**0.14**

*IQR* interquartile range, *SEM* standard error of the mean, *SD* standard deviation, *yrs* years, *n* number.

Bold values are statistically significant for *p* = 0.05; significance level was set at 5%.

At baseline, men were significantly older than women, showed significantly higher median PRL levels, higher mean BMI, and higher prevalence of impaired BMD, and presented predominantly with headache. Of the 28% men with impaired BMD, 21% suffered from osteopenia and 7% from osteoporosis, whereas the 2% of women with impaired BMD all suffered from osteopenia and none fulfilled the criteria for osteoporosis. Macroprolactinomas and cavernous sinus infiltration were more commonly seen in men than in women. Secondary adrenal deficiencies were noted significantly more often in men, whereas secondary hypothyroidism and gonadotropin deficiency were not significantly different between men and women. A primary surgical approach was performed in 41 women (68%) and in 12 men (30%).

### Early results

At 4 ± 2.9 months (± SD), serum PRL values had decreased significantly in both cohorts, from 1979 μg/L (IQR 768–6875) to 68 μg/L (IQR 12–282, *p* < 0.001) in men and from 152 μg/L (IQR 89–268) to 15.2 μg/L (IQR 7–51, *p* < 0.001) in women. Overall, serum PRL values remained significantly higher in men than in women (*p* = 0.001). PRL values were in the normal range in 68% of women versus 36% of men (*p* = 0.002). At early follow-up, serum PRL levels remained significantly higher in patients with impaired baseline BMD (126 μg/L; IQR, 20–755) compared to those with normal baseline BMD (19 μg/L; IQR, 7–79; *p* = 0.05).

### Long-term results

The median long-term follow-up was 79 months (range 13–408) and was not significantly different between the sexes (*p* = 0.14).

**Figure 1:**
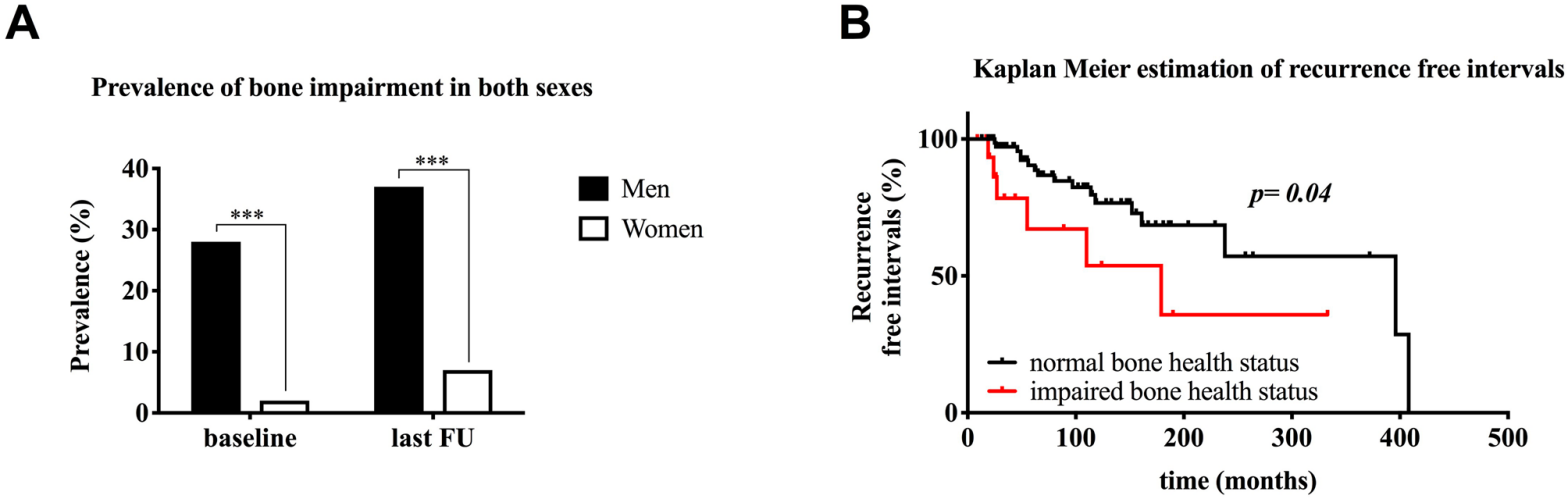
(**A**) Prevalence of bone impairment in both sexes. Significantly more men with prolactinomas suffered from bone impairment, both at baseline (28 vs*.* 2%, *p* < 0.001) and at last follow-up (37 vs*.* 7%, *p* < 0.001), compared to women. (**B**) Kaplan–Meier estimation of recurrence-free intervals. The median (± SD) recurrence-free intervals were significantly shorter in patients with impaired BMD (179 ± 72 months) than in those with normal BMD (396 ± 117 months; log-rank test, *p* = 0.04).

At last follow-up, impaired BMD was recorded in 37% of men and 7% of women (*p* < 0.001; Fig. 1A).

At this time point, 26% of men and 2% of women suffered from osteopenia, whereas 11% of men and 5% of women suffered from osteoporosis (Table 2).

**Table 2:** Characteristics of patients with impaired bone mineral density.

Patient no	Sex	Cohort	Bone status baseline	Bone status long-term FU	Hydrocortisone repl	B repl	V/C repl	Testosterone/estrogen repl	PRL levels baseline	PRL levels first FU	PRL levels long-term FU	Hypogonadismlast follow-up
1	M	Med	OP (S,F)	Normal	No	No	No	No	9155	93.4	13.8	No
2	M	Med	OP (S,F)	OP (S,F)	Yes	No	Yes	Yes	19,200	2.1	4.5	No
3	M	Med	OO (S,F,T)	OO (S,F,T)	No	No	No	Yes	41,920	50.4	40.7	No
4	M	Med	OP (S,F,T)	OP (S,F,T)	No	No	Yes	No	4422	6.6	2.8	No
5	M	Surg	OP (S,F,T)	OO (S,F,T)	No	Yes	Yes	Yes	6473	877	0.5	No
6	M	Med	OP (S,F), OO (T)	OP (S,F), OO (T)	No	No	Yes	Yes	31,940	918	172	No
7	M	Med	OP (S,F,T)	OP (S,F,T)	No	No	Yes	Yes	29,687	418.9	195.9	No
8	M	Med	OO (S), OP (F,T)	OO (S), OP (F,T)	No	No	YES	Yes	791	63.2	12.8	Yes
9	M	Med	OP (T)	OP (T)	No	No	Yes	Yes	12,480	857	10.4	Yes
10	M	Med	OP (S,T)	OP (S,T)	No	No	Yes	Yes	1510	447.5	22.8	Yes
11	M	Surg	OP (S,F,T)	OP (S,F,T)	No	No	No	Yes	79.7	9.4	24.8	No
12	M	Surg	Normal	OP (n)	No	No	Yes	No	130	76.8	11.9	No
13	M	Med	Normal	OP (S,F)	No	No	Yes	No	1718	112.4	59.8	No
14	M	Surg	Normal	OP (n)	No	No	No	No	1080	12.3	11.7	No
15	M	Med	Normal	OP (S,F,T)	No	No	Yes	Yes	9160	231	29.4	No
16	F	Med	OP (S)	OP (S)	No	No	No	Yes	83.4	158.7	n/a	No
17	F	Med	Normal	OO (S,F,T)	No	no	Yes	No	105	34	7	No
18	F	Surg	Normal	OO (S)	No	No	Yes	Yes	465	11.3	48.3	Yes
19	F	Surg	Normal	OO (S)	No	No	Yes	Yes	70.4	17.1	17	No

*OP* osteopenia, *OO* osteoporosis, *S* lumbar spine, *F* femoral bone, *T* tibia, *n* no specifications, *repl*. Replacement, *B/V/C* bisphosphonate/vitamin D/calcium, *FU* follow-up, *med* medical, *surg* surgical, *M* male, *F* female.

Compared to baseline, there was no significant increase in the prevalence of impaired BMD in men (28% vs. 37%, *p* = 0.47) or in women (2% vs. 7%, *p* = 0.21) and the prevalence of both osteopenia (9% vs .12%, *p* = 0.66) and osteoporosis (3% vs. 7%, *p* = 0.17) showed a non-significant increase. No pathological fractures were documented in our patient cohort. While the overall prevalence of impaired BMD (i.e., in both men and women) was 12% at baseline, it had increased to 19% by the last follow-up (*p* = 0.24), independent of the primary treatment strategy; i.e. surgical (4% *vs* 12%, *p* = 0.16) or medical (21% vs .27%, *p* = 0.63) treatment.

The prevalence of bone impairment at last follow-up was significantly higher in patients with persistent hyperprolactinemia than those with normoprolactinemia (42% vs. 15%; *p* = 0.04); in hypogonadal compared with eugonadal patients (33% vs. 10%; *p* = 0.01); and in patients with persistent sex hormone therapy compared to those without (46% vs. 10%; *p* < 0.001). There was no significant increase in the prevalence of bone impairment in patients without DA agonist therapy compared to those with persistent need for DA agonists (16% vs. 20%; *p* = 0.79). Regarding the adenoma size, the prevalence of bone impairment was significantly greater in patients with macroadenomas than in patients with microadenomas at baseline (19% vs. 4%, *p* = 0.03), but not at last follow-up (26% vs. 11%, *p* = 0.07).

Total testosterone levels in men significantly increased, namely form 5.9 ± 4.8 nnoml/l at baseline to 13.3 ± 3.6 nmol/l in the long-term (*p* = 0.001). Likewise, estradiol levels in women significantly increased, from 62 ± 68 pg/ml at baseline to 161 ± 371 pg/ml in the long-term (*p* = 0.003).

The duration of clinical symptom onset reported prior to diagnosis was 18 ± 69 months (± SD). The calculated duration of hyperprolactinemia and hypogonadism was 41 ± 82 months and 38 ± 98 months, respectively. Thereby, the duration of hyperprolactinemia in patients with impaired bone density was greater over the long-term than in patients with normal bone density, although this was not significant (33 ± 100 months vs. 86 ± 90 months, *p* = 0.24). Similarly, the duration of hypogonadism was greater in patients with impaired bone density than in those with normal BMD, although this was not significant (104 ± 84 months vs. 38 ± 81 months, *p* = 0.15). In particular, there was no significant difference between the sexes in the time that patients remained hypogonadal; this measured 40 ± 66 months in men versus 33 ± 114 months in women (*p* = 0.29).

In patients with resolution of hyperprolactinemia, the time to performance of bone densitometry was 47 ± 64 months, with a longer time period in men than in women, although this was not statistically significant (31 ± 58 months vs. 57 ± 67 months; *p* = 0.06).

PRL levels had normalized in most patients by the long-term follow-up, independent of gender (men vs. women; 82% vs. 89%, *p* = 0.37). Nevertheless, significantly fewer women required DA agonists for the long-term control of hyperprolactinemia than men (75 vs. 42%, *p* = 0.001). Also, PRL levels had normalized independent of the primary treatment approach (surgery vs. DAs; 92% vs. 80% *p* = 0.14). We noted that significantly fewer patients in the surgical versus medical cohort required DA agonists over the long-term (32% vs. 79%, *p* < 0.001). Gonadotropin deficiency significantly improved both in men and women (same *p* < 0.001), as did headache (*p* < 0.001 and *p* = 0.02, respectively). Secondary hypothyroidism and secondary adrenal insufficiency improved in both groups, although not significantly. In 41 (68%) premenopausal women with available data confirming amenorrhea at baseline, no significant association between duration of amenorrhea and long-term BMD status was noted (r = 0.20, *p* = 0.08). In addition, the duration of amenorrhea in women was not a risk factor for impaired BMD at last follow-up (OR 1.0, 95%CI 1.0–1.1; *p* = 0.32). Furthermore, the amount of time between resolution of hyperprolactinemia and the performance of bone densitometry was not a risk factor for impaired BMD at last follow-up (OR 1.0, 95% CI 1.0–1.1, *p* = 0.14).

Of the 100 patients assessed with DXA, only one patient with osteopenia at baseline was noted with a normal BMD over the long-term (patient number 1, Table 2). While a normal BMD status was noted both at baseline and at last follow-up in 82 patients, seven of those patients with initially normal BMD demonstrated impaired BMD over the long-term. In addition, in eight patients osteopenia was noted both at baseline and over the long-term, as was osteoporosis in two further patients. Thus, persistent bone impairment in patients with prolactinomas was common, despite long-term control of hyperprolactinemia and hypogonadism in the majority of them. As a result, of the 12 patients with low bone density at baseline (OP and OO), 11 also had low BMD in the long-term, and deterioration was noted in an additional 7 patients.

At last follow-up, recurrence of prolactinoma was observed in 35% of patients with an impaired BMD compared to 22% of patients with a normal BMD (*p* = 0.35). Specifically, the recurrence rate was 36% in men and 33% in women with an impaired BMD, and 17% in men vs. 24% in women with normal BMD (*p* = 0.76). In addition, recurrence of a prolactinoma was noted in 20% of patients with upfront surgery compared to 30% of patients treated with DAs (*p* = 0.25). There was no significant difference in the recurrence-free intervals of prolactinomas between men and women (178 ± 18 months vs. 288 ± 28 months; log-rank test, *p* = 0.25). However, the median (± SD) recurrence-free intervals were significantly shorter in patients with impaired BMD (179 ± 72 months) than in those with normal BMD (396 ± 117 months; log-rank test, *p* = 0.04, Fig. 1B).

The risk factors associated with long-term bone impairment are summarized in Table 3. Significant risk factors in the univariable analysis were: patient age, male sex, persistent hyperprolactinemia including length of hyperprolactinemia, and persistent hypogonadism. The multivariable logistic regression revealed male sex (OR 16.4, 95% CI 2.4–114.3, *p* = 0.01) and persistent hyperprolactinemia (OR 5.6, 95% CI 1.0–32.5, *p* = 0.05), but not persistent hypogonadism (OR 3.1, 95% CI 0.8–12.4, *p* = 0.12) or primary treatment strategy (OR 1.2, 95% CI 0.3–5.2, p = 0.81) as independent risk factors for long-term bone impairment (Table 3).

**Table 3:** Risk factors for impaired BMD at last follow-up in patients with prolactinomas.

Risk factors for iBMD at last FU	Univariable analyses OR (95% CI)	*p* value	Multivariable analyses OR (95% CI)	*p* value
Age, years	1.1 (1.0–1.1)	**0.01**	1.0 (1.0–1.1)	0.66
Sex: men	8.0 (2.4–26.9)	**0.001**	16.4 (2.4–114.3)	**0.01**
Primary medical approach	2.8 (1.0–8.2)	0.06	1.2 (0.3–5.2)	0.81
BMI at baseline	1.0 (0.9–1.1)	0.46		
Tumor size (e.g., macroadenoma)	2.8 (0.9–8.6)	0.07	2.0 (0.4–10.9)	0.41
BMI at last FU	1.0 (0.9–1.1)	0.86		
Persistent need for DA-agonists	1.3 (0.5–3.7)	0.61		
Persistent hyperprolactinemia	4.2 (1.2–15.5)	**0.03**	5.6 (1.0–32.5)	**0.05**
Persistent hypogonadism	4.8 (1.6–14.4)	**0.006**	3.1 (0.8–12.4)	0.12
Follow-up time, months	1.0 (0.9–1.0)	0.17		
Length of hypogonadism	1.0 (1.0–1.1)	0.15		
Length of hypperprolactinemia	1.0 (1.0–1.1)	0.26		

*BMI* body mass index, *DA* dopamine, *FU* follow-up, *iBMD* impaired bone mineral density.

Bold values are statistically significant for *p* = 0.05; significance level was set at 5%.

### Morbidity and mortality

There was no mortality in either cohort. Postoperative complications in the surgical group consisted of transient rhinoliquorrhea (3%), syndrome of inappropriate antidiuretic hormone (SIADH) secretion (12%), and diabetes insipidus (13%). In the medical group, prolonged nausea occurred in 9% of patients, dopamine agonist-induced impulse control disorders were observed in two men (4%)^23^, and vertigo in 3% of patients with no difference between men and woman.

## Discussion

This large prolactinoma cohort study shows that: (1) although both hyperprolactinemia and hypogonadism are under control in the majority of patients at a median follow-up of ≈ 7 years, the prevalence of bone impairment was and continues to be significantly higher in men than in women; (2) persistent hyperprolactinemia and male sex, but not persistent hypogonadism, are independent risk factors for long-term bone impairment in prolactinoma patients; and (3) recurrence-free intervals are significantly shorter in prolactinoma patients with impaired BMD.

### Long-term impact of prolactinoma treatment on bone mineral density

Hyperprolactinemia and the associated hypogonadism affect bone turnover in prolactinoma patients^10, 14, 15^. While age-related bone loss might have contributed to bone fragility over our study period of almost seven years to some extent^24, 25^, long-lasting hyperprolactinemia has been found to be a major contributor to bone impairment, even when hyperprolactinemia is brought under control^15, 20^, corroborating our results. Consistently, treatment with DA agonists over 2 years was not found to restore bone impairment in young patients suffering from hyperprolactinemia^12^.

We further noted that significantly more men than women suffered from bone impairment at study entry.

While amenorrhea in women is easily detected and investigated, men often do not report the more non-specific symptoms of hypogonadism, such as loss of libido. Consequently, women probably suffer from hyperprolactinemia and hypogonadism over a much shorter period before diagnosis, and treatment is initiated much earlier than for men^26^. This hypothesis is further supported by the current finding that the age at diagnosis was significantly higher for men than for women. Likewise, macroprolactinomas were more frequently encountered in men than in women, possibly contributing to both the higher baseline PRL levels as well as the subsequent higher prevalence of bone impairment in men compared to women. Namely, initial prolactin levels and the size of the tumor may reflect how long the disease has been present, given that bone loss has been associated with the duration of amenorrhea in women with prolactinomas^8^. Nevertheless, in this study cohort, the duration of therapy or the duration of amenorrhea in women was not a significant risk factor for BMD development. Furthermore, treatment of the prolactinoma might interfere with BMD development. Conversely, while couldn’t observe a difference in testosterone replacement, vitamin D supplementation, or the use of hydrocortisone in men versus women, it is conceivable that a certain selection bias towards the screening of osteoporosis in more severely affected men with prolactinoma took place at study entry, with 3:2 ratio of women to men. This may partly be explained by the fact that the prevalence of prolactinoma is known to be higher in women than in men^27, 28^. In addition, although health insurance in Switzerland covers medical investigation and therapy, decisions regarding whether to screen for bone density are not based on financial considerations. Bone measurement and programs for osteoporosis prevention have mainly focused on post-menopausal women^8, 9^, while this condition often remains underdiagnosed in men^10–12^. Consistently, in a large study cohort, significantly fewer men received evaluation for osteoporosis following a distal radial fracture, with rates of evaluation unacceptably low according to published guidelines^12^.

In the context of prolactinomas, the need for awareness of bone loss in both sexes might thus have been underestimated in men, with those affected more severely being preferentially assessed. Thus, screening of bone loss in both sexes should not be underestimated in prolactinoma patients, regardless of the primary treatment chosen (i.e., surgical or medical), as the primary treatment did not seem to influence the prevalence of bone impairment in our cohort.

### Recurrence rates of prolactinomas

We noted no differences in the recurrence rates between men and women after DA agonist withdrawal, whereas other authors reported more recurrences in men than women^29^, possibly because men suffer more often from macroprolactinomas than women do^30^. While recurrence-free intervals were not significantly different with regard to adenoma size, patients with impaired BMD had significantly shorter recurrence-free intervals than those with normal BMD. This is an intriguing finding. It is conceivable that the smaller sample size of patients with macroadenomas conceals a true effect^31^. Indeed, macroprolactinomas in men are associated with longer lasting hyperprolactinemia and related hypogonadism, with subsequently impaired BMD^11, 32^. Nevertheless, the adenoma size per se might not be the only factor that determines the severity of the disease. In contrast, impaired BMD, which as “end organ” reflects the full range of the disease, including duration of hypogonadism, might thus become a more comprehensive surrogate marker for the severity of long-lasting hyperprolactinemia. Given that osteoporosis prevention has particularly focused on postmenopausal women (with prolactinomas), assessment of BMD in men with prolactinomas might become routine and incorporated into study guidelines. Further studies should be directed at how to improve bone health in prolactinoma patients in general and how to better evaluate patients at risk at the earliest time point possible.

## Study limitations

This study suffers from the limitations of any retrospective study, and of the single-center design. In 83 of 100 patients, data was available on the onset of symptoms prior to diagnosis. Thus, the duration of hypogonadism and hyperprolactinemia, or the time period between resolution of hyperprolactinemia and the performance of bone densitometry, could not be retrieved for all patients. In addition, it reflects an approximate estimation of the duration of both hypogonadism and hyperprolactinoemia. In addition, a true effect for the association between amenorrhea duration and long-term BMD status might have been concealed given the sample size of premenopausal women with available data confirming amenorrhea at baseline.

Given that there was no prospective assessment of DA-induced impulse control disorders, the true number of patients experiencing them might be underestimated. Likewise, although severe personality changes have been reported, these might often not be mentioned by the patients due to feelings of shame^12^.

No treatments with growth hormones (GH) were noted in this cohort, and not all patients werescreened for growth hormone (GH) deficiency using validated dynamic testing, or for vitamin D concentrations and active smoking status, so it is possible that these parameters influenced the bone health status in some patients^33–38^. Patients with osteopenia and osteoporosis have been grouped together as patients with impaired BMD, and statistical uncertainty in this sample size precluded us from discriminating between osteopenia and osteoporosis in both men and women. Numeric BMD values in this patient cohort are missing, thus quantification of bone improvement following treatment of hyperprolactinoma and hypogonadism was not possible. Allocation into groups (i.e. normal, osteopenia, osteoporosis) indirectly reflects changes in bone impairment. This pooling doesn’t incorporate the fact that osteopenia is present in about 15% of young, healthy women^39^. Likewise, using multiple logistic regression analysis to assess independent predictors influencing BMD—such as location of BMD measurement, testosterone replacement, vitamin D supplementation, and use of hydrocortisone (see Table 2)—was not statistically feasible. In addition, the location used for BMD measurement with DXA was not consistent in all patients examined. Although there is a significant correlation for BMD values between anatomical regions such as the spine, proximal femur and forearm, the validity of DXA measurement in prolactinoma patients favors the spine only, as data show that femoral BMD measurement might mask BMD effects exerted by hyperprolactinemia and associated hypogonadism^8, 40^.

Our biochemical definition of persistent hypogonadism (i.e. inadequate gonadotropins in the presence of low estradiol) may have underestimated a true association between persistent hypogonadism and long-term BMD status, as it doesn’t incorporate those women with sporadic normal estradiol levels at follow-up, but ongoing oligomenorrhea.

## Conclusions

The prevalence of bone impairment is and continues to be significantly higher in men with prolactinomas than in women. Impaired BMD as “end organ” reflects the full range of the disease and could become a surrogate marker for the severity of long-term hyperprolactinemia and associated hypogonadism.

## Methods

This retrospective cohort study included all consecutive prolactinoma patients with osteodensitometric data at study entry and at long-term follow-up (> 12 months) who were treated at our tertiary referral center between 1997 and 2015. All patients fulfilled the diagnostic criteria of a prolactin (PRL)-secreting pituitary adenoma (i.e., PRL levels > 30 µg/L without evidence of pituitary stalk compression, primary hypothyroidism or drug-induced hyperprolactinemia), and had a positive pituitary magnetic resonance imaging (MRI) scan. The indication for first-line pituitary surgery was local complications of the adenomas or the patient’s preference to undergo surgery rather than long-term DA agonist therapy, as reported previously^11, 41^. Each patient’s situation and primary treatment were discussed at the interdisciplinary pituitary tumor board meeting.

BMD was assessed by dual-energy X-ray absorptiometry (DXA, HOLOGIC, Bedford, MA, USA) at the femoral bone and/or spine at baseline and at last follow-up. A T-score ≥ 1 SD was regarded as normal, whereas a T-score of − 1.5 to − 2.5 SD suggested osteopenia, and ≤ −2.5 SD suggested osteoporosis. The Z-score was used in the diagnosis of impaired BMD in premenopausal women and in men aged < 50 years^42, 43^. Impaired BMD was considered in patients with osteopenia and/or osteoporosis^11, 44^.

MRI was performed on a 1.5-T or 3-T system including a Proton/T2-weighted whole-brain study with unenhanced, contrast-enhanced, dynamic contrast-enhanced and post contrast-enhanced overlapping studies in the axial, sagittal and coronal planes over the sellar region^45, 46^. A tumor with a diameter of 1–10 mm was defined as a microadenoma, and a tumor > 10 mm in diameter was defined as a macroadenoma. Infiltration of the cavernous sinus was defined as ≥ two-thirds encasement of the internal carotid artery by the adenoma, as previously described^47, 48^.

Patient characteristics recorded at study entry included age, body mass index (BMI), co-occurring clinical symptoms such as headache, pituitary axes deficits and radiological findings. Symptoms such as galactorrhea and amenorrhea in women or infertility and/or lack of libido or erectile dysfunction in men were noted separately. Partial hypopituitarism was defined as impaired secretion of one or more pituitary hormones. PRL levels were assessed. These included the immunoradiometric PRL assay with serum dilution in order to overcome the high-dose PRL hook effect^49–52^. Secondary adrenal insufficiency was noted in the presence of low cortisol levels in the serum or in cases where cortisol level was normal but responses to the adrenocorticotropic hormone (ACTH) stimulation test or insulin tolerance test were inadequate. The diagnosis of secondary hypothyroidism was made in the presence of low-normal thyrotropin (TSH) levels and a low free thyroxin (FT4) level. Hypogonadotropic hypogonadism, or central hypogonadism, leads to secondary amenorrhea or irregular menstrual cycle in female patients and impaired libido in males. Biochemically, inadequately normal-low gonadotropins can be documented, resulting in lack of production of estradiol or testosterone^53, 54^.

For men, two fasting measurements of total testosterone concentrations were used for the screening for androgen deficiency^55, 56^. Blood samples were collected after overnight fasting. Serum concentrations of total testosterone (normal reference range, 9.9–28.0 nmol/L) were measured using the Elecsys-System (ROCHE diagnostics, Rotkreuz, Switzerland) as well as the Centaur-System (BAYER diagnostics, Zürich, Switzerland)^57^. To evaluate the day-to-day variance, total testosterone was measured by the Elecsys-System on two different days within one month at 8 am in the fasting state^58^.

In order to estimate the duration of hyperprolactinemia and subsequent hypogonadism, we reviewed patients’ records in order to assess the reported onset of clinical symptoms prior to diagnosis (i.e., onset of galactorrhea/amenorrhea in women; loss of libido or erectile dysfunction in men). The estimated duration of hyperprolactinemia and hypogonadism was then calculated from the date of reported onset of symptoms to the date of laboratory correction of hyperprolactinemia or hypogonadism during the follow-up visit.

Pituitary surgery (n = 53) was performed using a transseptal, transsphenoidal microsurgical approach, as described previously^45, 59^. Postoperatively, body weight, fluid intake and output, serum electrolytes, and serum and urine osmolality were monitored daily. An antibiotic was administered in the perioperative setting and discontinued after 24 h.

Early follow-up took place about three months after surgery or at the initiation of DA agonist treatment. The dose of the DA agonist was increased if PRL levels were still elevated (> 30 µg/L) in the medical cohort. If patients in the surgical cohort had elevated PRL at pathological levels, DA agonist therapy was initiated.

A standardized protocol was followed for the withdrawal of DA agonists. In the medical cohort, DA agonists were tapered 24 months after initiation of the medical therapy if PRL levels had normalized and tumor reduction of > 50% was attained at the time of radiological follow-up, as defined previously^60, 61^. Recurrence was defined as an increase in PRL levels above the normal range (> 25 µg/L for women, > 20 µg/L for men) during the last follow-up period after a previous remission, irrespective of radiological findings^62, 63^.

### Statistical analysis

Data were analyzed using IBM SPSS statistical software Version 24.0 (IBM Corp., New York, NY, USA) and visualized using GraphPad Prism (V7.03 software, San Diego, CA, USA). Continuous variables were examined for homogeneity of variance and are expressed as mean ± SD except where otherwise noted. PRL levels are presented as median values and interquartile range (IQR, 25th–75th percentile). For comparisons of means between two groups, Student’s t-test was used for normally distributed data, and the Mann–Whitney test for nonparametric data. The Wilcoxon signed-rank test was used to evaluate paired differences in PRL, testosterone and estradiol levels before and after treatment^64^. Categorical variables were compared using Pearson’s chi-square test or Fisher’s exact test, as appropriate^65^. The Kaplan–Meier method was used to analyze recurrence-free intervals during follow-up, and significance was calculated using the log-rank (Mantel–Cox) test. To identify potential associations with impaired BMD at last follow-up, possible risk factors (patient age, sex, primary therapeutic approach, BMI, initial tumor size [i.e. macroadenoma], persistent need for DA agonists, persistent hyperprolactinemia and hypogonadism) were included, and multivariate analysis was performed with a binary logistic regression model. OR and 95% CI were calculated and *p* values ≤ 0.05 were considered statistically significant^66, 67^.

### Ethical standards and patient consent

All methods were performed in accordance with the relevant guidelines and regulations of Scientific Reports. The study is a retrospective data project using existing data to evaluate registry data quality, and there was no any patient contact for the study, therefore there was no patient consent process. The Human Research Ethics Committee of Bern (Kantonale Ethikkommision KEK Bern, Bern, Switzerland) approved the project (KEK no. 10-10-2006 and 8-11-2006). The ethics commit waived the need for informed consent for this study as part of the study approval. The study was performed in accordance with the ethical standards laid down in the 1964 Declaration of Helsinki and its later amendments.

## Data Availability

The authors agree to share data on request.

## References

[1] Gourlay ML (2012). Bone-density testing interval and transition to osteoporosis in older women. N. Engl. J. Med..

[2] Nojiri S (2019). Comorbidity status in hospitalized elderly in Japan: analysis from National Database of Health Insurance claims and specific health checkups. Sci Rep.

[3] de Lima CAD (2019). Postmenopausal osteoporosis reference genes for qPCR expression assays. Sci. Rep..

[4] Ebeling PR (2008). Clinical practice. Osteoporosis in men. N. Engl. J. Med..

[5] Dirschl, D. R. Shame on Us! Men Need Osteoporosis Care, Too! Commentary on an article by Carl M. Harper, MD, et al.: "Distal Radial Fractures in Older Men. A Missed Opportunity?". *J. Bone Joint Surg. Am.* **96**, e186. 10.2106/JBJS.N.00792 (2014). 10.2106/JBJS.N.00792 25378519

[6] Harper CM, Fitzpatrick SK, Zurakowski D, Rozental TD (2014). Distal radial fractures in older men: a missed opportunity?. J. Bone Joint Surg. Am..

[7] Di Somma C (1998). Bone marker and bone density responses to dopamine agonist therapy in hyperprolactinemic males. J. Clin. Endocrinol. Metab..

[8] Naliato EC (2008). Bone density in women with prolactinoma treated with dopamine agonists. Pituitary.

[9] Mazziotti G, Frara S, Giustina A (2018). Pituitary diseases and bone. Endocr. Rev..

[10] Iacovazzo D, De Marinis L (2014). Treatment of hyperprolactinemia in post-menopausal women: pros. Endocrine.

[11] Andereggen L (2016). Long-term follow-up of primary medical versus surgical treatment of prolactinomas in men: effects on hyperprolactinemia, hypogonadism and bone health. World Neurosurg..

[12] Colao A (2000). Prolactinomas in adolescents: persistent bone loss after 2 years of prolactin normalization. Clin. Endocrinol..

[13] Handa K (2019). Bone loss caused by dopaminergic degeneration and levodopa treatment in Parkinson's disease model mice. Sci. Rep..

[14] Mazziotti G (2011). Vertebral fractures in males with prolactinoma. Endocrine.

[15] Mazziotti G (2011). High prevalence of radiological vertebral fractures in women with prolactin-secreting pituitary adenomas. Pituitary.

[16] Shibli-Rahhal A, Schlechte J (2009). The effects of hyperprolactinemia on bone and fat. Pituitary.

[17] Faje AT, Klibanski A (2015). The treatment of hyperprolactinemia in postmenopausal women with prolactin-secreting microadenomas: cons. Endocrine.

[18] D'Sylva C, Khan T, Van Uum S, Fraser LA (2015). Osteoporotic fractures in patients with untreated hyperprolactinemia vs. those taking dopamine agonists: a systematic review and meta-analysis. Neuro Endocrinol. Lett..

[19] Bolanowski M, Jawiarczyk-Przybylowska A, Halupczok-Zyla J (2015). Osteoporosis in pituitary diseases: lessons for the clinic. Expert Rev. Endocrinol. Metab..

[20] Seriwatanachai D, Krishnamra N, van Leeuwen JP (2009). Evidence for direct effects of prolactin on human osteoblasts: inhibition of cell growth and mineralization. J. Cell. Biochem..

[21] Iacovazzo D, De Marinis L (2015). Treatment of hyperprolactinemia in post-menopausal women: pros. Endocrine.

[22] Pekic S, Medic Stojanoska M, Popovic V (2019). Hyperprolactinemia/prolactinomas in the postmenopausal period: challenges in diagnosis and management. Neuroendocrinology.

[23] Dogansen SC (2019). Dopamine agonist-induced impulse control disorders in patients with prolactinoma: a cross-sectional multicenter study. J. Clin. Endocrinol. Metab..

[24] Kyvernitakis I (2013). The effect of age, sex hormones, and bone turnover markers on calcaneal quantitative ultrasonometry in healthy German men. J. Clin. Densitom..

[25] Sinnesael M, Boonen S, Claessens F, Gielen E, Vanderschueren D (2011). Testosterone and the male skeleton: a dual mode of action. J. Osteoporos..

[26] Daly AF (2006). High prevalence of pituitary adenomas: a cross-sectional study in the province of Liege, Belgium. J. Clin. Endocrinol. Metab..

[27] Vroonen L, Daly AF, Beckers A (2019). Epidemiology and management challenges in prolactinomas. Neuroendocrinology.

[28] Fontana E, Gaillard R (2009). Epidemiology of pituitary adenoma: results of the first Swiss study. Rev. Med. Suisse.

[29] Colao A (2007). Predictors of remission of hyperprolactinaemia after long-term withdrawal of cabergoline therapy. Clin. Endocrinol..

[30] Barber TM (2011). Recurrence of hyperprolactinaemia following discontinuation of dopamine agonist therapy in patients with prolactinoma occurs commonly especially in macroprolactinoma. Clin. Endocrinol..

[31] Button KS (2013). Power failure: why small sample size undermines the reliability of neuroscience. Nat. Rev. Neurosci..

[32] De Rosa M (2003). Hyperprolactinemia in men: clinical and biochemical features and response to treatment. Endocrine.

[33] Chiloiro S (2018). Prevalence of morphometric vertebral fractures in "difficult" patients with acromegaly with different biochemical outcomes after multimodal treatment. Endocrine.

[34] Mazziotti G (2013). Vertebral fractures in patients with acromegaly: a 3-year prospective study. J. Clin. Endocrinol. Metab..

[35] Li H (2020). Smoking-induced risk of osteoporosis is partly mediated by cadmium from tobacco smoke: the MrOS Sweden Study. J. Bone Miner. Res..

[36] Strozyk D, Gress TM, Breitling LP (2018). Smoking and bone mineral density: comprehensive analyses of the third National Health and Nutrition Examination Survey (NHANES III). Arch. Osteoporos..

[37] Burt LA (2019). Effect of high-dose vitamin D supplementation on volumetric bone density and bone strength: a randomized clinical trial. JAMA.

[38] Andereggen L, Frey J, Christ E (2020). Long-term IGF-1 monitoring in prolactinoma patients treated with cabergoline might not be indicated. Endocrine.

[39] Khan AA (2004). Standards and guidelines for performing central dual-energy X-ray absorptiometry in premenopausal women, men, and children. J. Clin. Densitom..

[40] Naliato EC (2005). Prevalence of osteopenia in men with prolactinoma. J. Endocrinol. Invest..

[41] Andereggen L (2016). 10-Year follow-up study comparing primary medical vs. surgical therapy in women with prolactinomas. Endocrine.

[42] Carey JJ (2009). Dual-energy X-ray absorptiometry diagnostic discordance between Z-scores and T-scores in young adults. J. Clin. Densitom..

[43] Carey JJ (2007). DXA-generated Z-scores and T-scores may differ substantially and significantly in young adults. J. Clin. Densitom..

[44] Makarov SN, Noetscher GM, Arum S, Rabiner R, Nazarian A (2020). Concept of a radiofrequency device for osteopenia/osteoporosis screening. Sci. Rep..

[45] Andereggen L (2012). Selective inferior petrosal sinus sampling without venous outflow diversion in the detection of a pituitary adenoma in Cushing's syndrome. Neuroradiology.

[46] Andereggen L (2019). Influence of inferior petrosal sinus drainage symmetry on detection of adenomas in Cushing's syndrome. J. Neuroradiol..

[47] Cottier JP (2000). Cavernous sinus invasion by pituitary adenoma: MR imaging. Radiology.

[48] Wu ZB (2008). Five years follow-up of invasive prolactinomas with special reference to the control of cavernous sinus invasion. Pituitary.

[49] Karavitaki N (2006). Do the limits of serum prolactin in disconnection hyperprolactinaemia need re-definition? A study of 226 patients with histologically verified non-functioning pituitary macroadenoma. Clin. Endocrinol..

[50] Fahie-Wilson M, Smith TP (2013). Determination of prolactin: the macroprolactin problem. Best Pract. Res. Clin. Endocrinol. Metab..

[51] Suliman AM, Smith TP, Gibney J, McKenna TJ (2003). Frequent misdiagnosis and mismanagement of hyperprolactinemic patients before the introduction of macroprolactin screening: application of a new strict laboratory definition of macroprolactinemia. Clin. Chem..

[52] Fahie-Wilson MN, John R, Ellis AR (2005). Macroprolactin; high molecular mass forms of circulating prolactin. Ann. Clin. Biochem..

[53] Meysing AU (2004). GNRHR mutations in a woman with idiopathic hypogonadotropic hypogonadism highlight the differential sensitivity of luteinizing hormone and follicle-stimulating hormone to gonadotropin-releasing hormone. J. Clin. Endocrinol. Metab..

[54] Reindollar RH, Novak M, Tho SP, McDonough PG (1986). Adult-onset amenorrhea: a study of 262 patients. Am. J. Obstet. Gynecol..

[55] Matsumoto AM, Bremner WJ (2004). Serum testosterone assays–accuracy matters. J. Clin. Endocrinol. Metab..

[56] Wang C, Catlin DH, Demers LM, Starcevic B, Swerdloff RS (2004). Measurement of total serum testosterone in adult men: comparison of current laboratory methods versus liquid chromatography-tandem mass spectrometry. J. Clin. Endocrinol. Metab..

[57] Christ-Crain M (2004). Comparison of different methods for the measurement of serum testosterone in the aging male. Swiss Med. Wkly..

[58] Vermeulen A, Verdonck L, Kaufman JM (1999). A critical evaluation of simple methods for the estimation of free testosterone in serum. J. Clin. Endocrinol. Metab..

[59] Seiler RW, Mariani L (2000). Sellar reconstruction with resorbable vicryl patches, gelatin foam, and fibrin glue in transsphenoidal surgery: a 10-year experience with 376 patients. J. Neurosurg..

[60] Wass JA (2006). When to discontinue treatment of prolactinoma?. Nat. Clin. Pract. Endocrinol. Metab..

[61] Colao A (2003). Withdrawal of long-term cabergoline therapy for tumoral and nontumoral hyperprolactinemia. N. Engl. J. Med..

[62] Qu X (2011). Surgical outcomes and prognostic factors of transsphenoidal surgery for prolactinoma in men: a single-center experience with 87 consecutive cases. Eur. J. Endocrinol..

[63] Raverot G (2010). Prognostic factors in prolactin pituitary tumors: clinical, histological, and molecular data from a series of 94 patients with a long postoperative follow-up. J. Clin. Endocrinol. Metab..

[64] Dwivedi AK, Mallawaarachchi I, Alvarado LA (2017). Analysis of small sample size studies using nonparametric bootstrap test with pooled resampling method. Stat. Med..

[65] Amiri S, Modarres R (2017). Comparison of tests of contingency tables. J. Biopharm. Stat..

[66] Bursac Z, Gauss CH, Williams DK, Hosmer DW (2008). Purposeful selection of variables in logistic regression. Source Code Biol. Med..

[67] Mickey RM, Greenland S (1989). The impact of confounder selection criteria on effect estimation. Am. J. Epidemiol..

